# The evaluation of Hashimoto's thyroiditis with event-related potentials and magnetic resonance spectroscopy and its relation to cognitive function

**DOI:** 10.1038/s41598-021-82281-6

**Published:** 2021-01-28

**Authors:** Marta Waliszewska-Prosół, Joanna Bladowska, Sławomir Budrewicz, Marek Sąsiadek, Edyta Dziadkowiak, Maria Ejma

**Affiliations:** 1 Department of Neurology, Wroclaw Medical University, Wroclaw, Poland; 2 Department of General Radiology, Interventional Radiology and Neuroradiology, Wroclaw Medical University, Wroclaw, Poland

**Keywords:** Neuroscience, Endocrinology, Neurology

## Abstract

Thyroid dysfunction is very often accompanied by cognitive and affective disorders. The frequency of these disorders in patients with compensated Hashimoto’s thyroiditis (HT) is unknown. The aim of the present study was to evaluate brain dysfunction in euthyroid HT patients by means of event-related potentials (ERP) and magnetic resonance spectroscopy (MRS) and to correlate it with cognitive function. 68 patients with HT (59 female, 9 male) and 45 healthy controls were included in the study. All the patients underwent ERP including an analysis of N200 and P300 response parameters. MRS voxels were located in the posterior cingulate gyrus (PCG) and the left parietal white matter (PWM). The NAA/Cr, mI/Cr, and Cho/Cr ratios were analysed. The ERP parameters, MRS metabolite ratios and hormonal concentrations (TSH, fT3, fT4) as well as TGAb and TPOAb titer were also correlated. There was a significant prolongation of the latencies of N200 and P300 potentials and a significant decrease of P300 amplitude in HT patients than in the control group. There was a significant positive correlation between the mI/Cr ratio in the PCG area and P300 latencies. NAA/Cr ratio in the PCG region showed significant negative correlations with all N200 latencies. The results may suggest brain dysfunction in neurologically asymptomatic HT patients. ERPs undergo significant changes in patients with HT and may, in combination with MRS, constitute an important element in the recognition and monitoring of cognitive functions in this group of patients.

## Introduction

Hashimoto's thyroiditis (HT) is currently the most common autoimmune disease in humans^1^. Thyroid dysfunction is often accompanied by cognitive and affective disorders. The occurrence of cognitive disorders is particularly important, which are often overlooked, and can lead to the development of depression and have a great impact on the quality of life of patients^2,3^. Symptomatic clinically hypothyroidism can lead to changes in many cognitive domains, including attention, concentration, memory, and executive functions ^4,5^. Subclinical hypothyroidism may have a similar but more subtle effect on cognitive function^6^. However, there are few reports on cognitive disorders in patients with HT with compensated thyroid function, in whom difficulties with memory, focusing attention, or slowing down of thinking are often observed^7,8^. The cause of these symptoms has not yet been clarified.

One of the non-invasive and repeatable methods for assessing bioelectrical activity of non-specific areas of the brain associated with information processing processes is the study of cognitive event-related potentials (ERP). They allow us to objectify the clinical assessment of intellectual and cognitive functions^9,10^. In clinical practice, they allow to assess memory, decision-making and attention span processes^11–13^. ERPs are formed in extensive central nervous system neural networks, enabling assessment of the integrity and functional activity of the nerve pathways at the cortical and sub-cortical levels. Averaged cortical responses form a complex of components that correspond to the subsequent stages of recognition, analysis, and classification of the stimulus based on defined cognitive functions^11^. The most commonly used and best known in clinical practice is the potential of P300, also called by many researchers "*the wave of attention*"^14,15^. The method requires the active participation of the examined person and completing the task associated with a stimulus of a certain modality. It is believed that latency is a measure of the time spent on developing a stimulus and the amplitude indicates the size of the cognitive structures involved and corresponds to the subjective measure of the difficulty of the task^16^. The P300 wave itself arises when the problem is solved. The N200 wave, much less frequently evaluated and analysed in the literature, reflects the processes of initial identification and analysis of the stimulus. Most often it is an unconscious process and defined as readiness to solve a task^14,17^. One of the practical applications of ERP are attempts to use it as a diagnostic method in early diagnosis and assessment of the progress of the dementia process of various etiologies. Prolongation of P300 wave latency has been reported in patients with vascular or Alzheimer's dementia, Parkinson's disease, multiple sclerosis or metabolic encephalopathies^16,18^. Changes in ERP were also found in 42.5–56.6% of multiple sclerosis patients with cognitive impairment. They correlated with general disability and the duration of the disease. It has also been shown that depressive disorders are not only of cortical but also subcortical origin^19,20^.

Magnetic resonance (MR) is the method of choice in imaging of the brain. The advanced MR techniques empower the assessment of alterations which are far beyond the anatomical structure, searching deeper to discover any other variations almost at the cellular level. Magnetic resonance spectroscopy (MRS) has enabled the evaluation of certain metabolites in a variety of disorders involving the central nervous system (CNS). MRS is capable of detecting the changes in metabolite profiles in normal appearing white matter (NAWM) and normal appearing grey matter (NAGM)^21,22^. Accordingly, this advanced MR method may provide a potentially unique insight into the pathophysiology of cerebral changes associated with HT. Moreover, it should be stressed that in the available world literature there are no articles concerning the analysis of metabolic alterations within NAWM and NAGM in patients with HT apart from a single study by Bladowska et al.

The aim of the present study was to evaluate brain dysfunction in HT patients by means of ERP and MRS and to correlate it with cognitive function.

## Material and methods

The study comprised 68 patients with HT (59 women and 9 men, aged 20–63 years, mean 44.3) who met the criteria for the diagnosis of HT^1^. All the patients underwent ultrasound examination of the thyroid gland, were in the euthyreosis phase and treated with levothyroxine.

Patients with the presence of any neurological, psychiatric, cognitive, and autoimmune diseases, history of traumatic brain injury, underweight (BMI < 18.5 kg/m^2^) or obesity with a BMI of ≥ 35 kg/m^2^, defects in hearing, and administration of medications which change brain bioelectrical activity (e.g. neuroleptics, steroids, antiepileptic) were excluded. The control group (CG) consisted of 45 age and sex-matched healthy volunteers (39 women and 6 men, aged 22–64 years, mean 44.1). The exclusion criteria and the study protocol were the same in the CG.

The study protocol included a detailed neurological examination, with assessment of mental state using the Montreal Cognitive Assessment test (MoCA), Trail Making Test (TMT-A), Symbol Digit Modalities Test (SDMD) and Clock-Drawing Test (CDT) to screen for cognitive impairment, ERP and MRI examination. Laboratory tests included serum concentration of thyrotropin (TSH), free tri-iodothyronine (fT3), free thyroxine (fT4), and serum auto-antibodies against thyroid peroxidase (TPOAb) and thyroglobulin (TGAb) titers.

Approval for this research was given by the Commission of Ethics at the Wrocław Medical University (number of permission: KB-313/2013). Informed consent was obtained from each patient to participate in this study.

### Event related potentials protocol

Brain bioelectrical activity was recorded using superficial Ag/AgCl electrodes placed in the frontal region (Fz), central region (Cz) and parietal region (Pz), according to the international 10–20 system, with reference to linked earlobes and with a forearm ground. Impedance of all electrodes was maintained below 5 kΩ. ERP were elicited using the classic auditory “*oddball paradigm*”. The target tones (high frequency: 2000 Hz) occurred 20% of the time in each trial and non-target tones (low frequency: 1000 Hz) 80% of the time. Auditory stimuli were tones of 70 dB intensity and 200 ms duration. The patients were sitting in a comfortable position in a semi-darkened room and were asked to mentally count the stimuli. At least 30 target trials were averaged in each run and two runs were performed with each patient.

The responses were evaluated by means of a Nicolet 1000 Viking, with a 0.30/s, 70 Hz bandpass filter, sweep time 1000 ms and pre-stimulus baseline 250 ms. N200 was identified as a negative component with a latency of 180–320 ms and P300 was identified as a positive component with a latency of 280–450 ms after start of the stimulus. The latencies and amplitudes of N200 and P300 waves (“*peak to peak*”) were analysed. The procedure was compliant with the International Federation of Clinical Neurophysiology (IFCN) recommended standards^11,15^. We used the same methods as in our previous paper^18^.

### MR imaging

Imaging was performed with a 1.5 T SignaHdx MR scanner (GE Healthcare) using a head and neck 16-channel coil. The conventional MR sequences included: axial T1-weighted, axial, sagittal, and coronal T2-weighted images, FLAIR (fluid-attenuated inversion recovery sequence) and diffusion-weighted imaging (DWI) images. The protocol of magnetic resonance spectroscopy (MRS) examination used in our study was exactly the same as in previously published papers^21^. Post-processing of the images was performed using updated ReadyView software (GE Healthcare, ADW 4.6).

### Magnetic resonance spectroscopy protocol

**Figure 1:**
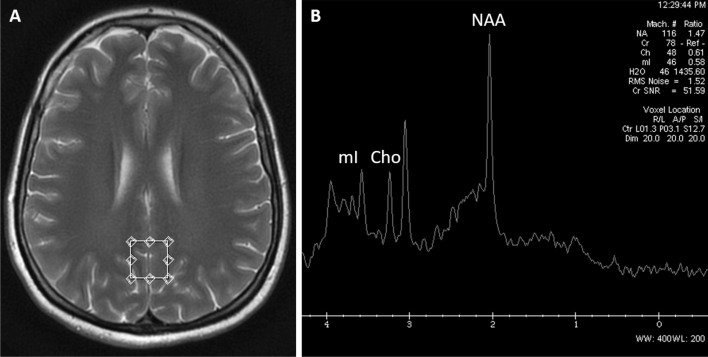
MR spectroscopy: axial T2-weighted image **(A)** showing a single voxel placed in the posterior cingulate gyrus region (PCG). The obtained spectrum **(B)** revealed a decrease in the NAA/Cr ratio in PCG. Figures source: Department of General and Interventional Radiology and Neuroradiology, Wroclaw Medical University; images performed with a with a 1.5 T SignaHdx MR scanner (GE Healthcare).

**Figure 2:**
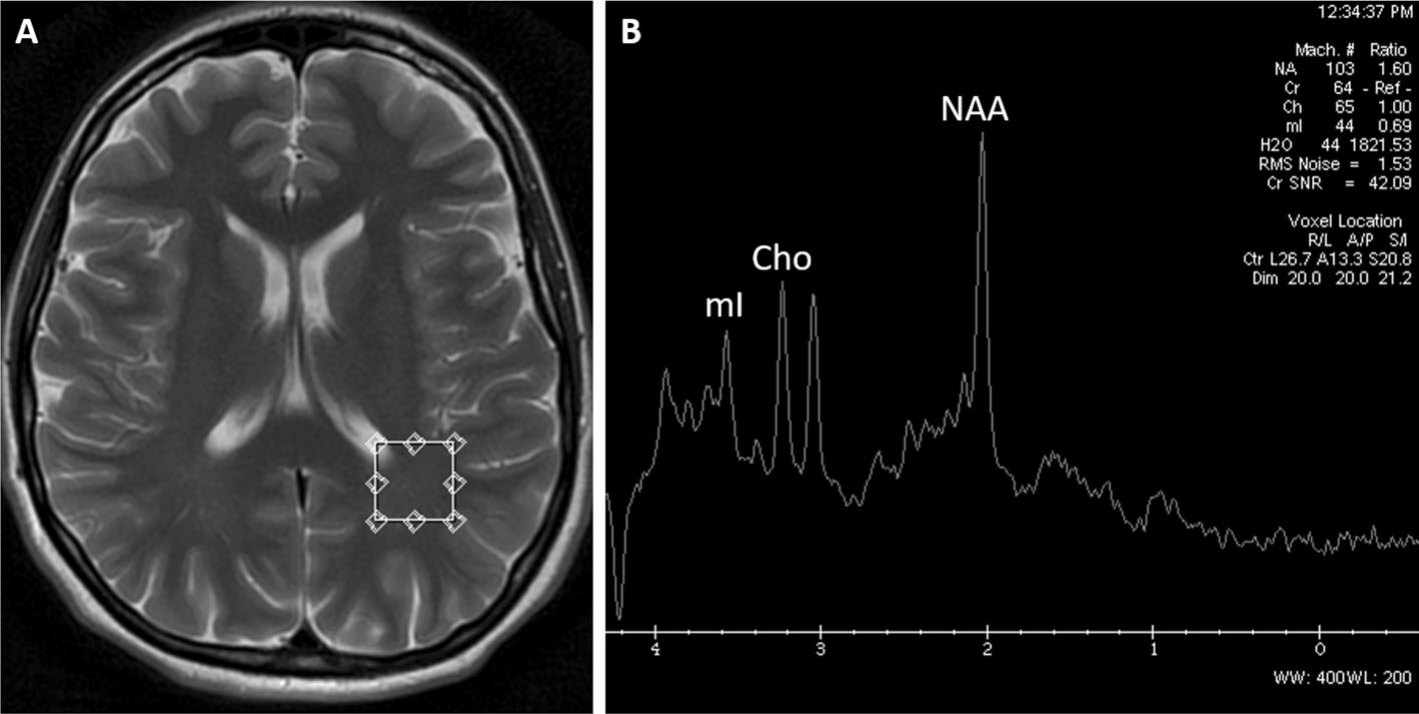
MR spectroscopy: axial T2-weighted image **(A)** presenting a single voxel placed in the left parietal white matter (PWM). The obtained spectrum **(B)** showed a decreased NAA/Cr ratio in PWM. Figures source: Department of General and Interventional Radiology and Neuroradiology, Wroclaw Medical University; images performed with a with a 1.5 T SignaHdx MR scanner (GE Healthcare).

The MRS examinations were conducted using the Single Voxel Spectroscopy (SVS) method (PRESS sequence) with the following acquisition parameters: TE = 35 ms, TR = 1500 ms, 128 acquisitions, number of excitations = 8. Using the localising axial T2-weighted images, voxels of 2 × 2 × 2 cm (8 cm^3^) size were placed in the normal appearing brain in 2 regions: the posterior cingulate gyrus cortex—PCG (Fig. 1) and the left parietal white matter—PWM (Fig. 2). The total acquisition time was 3 min 45 s for each voxel. MR spectroscopy studies were conducted by using the automated single-voxel MR spectroscopy package Proton Brain Examination/Single Voxel (PROBE/SV; GE Medical Systems, Milwaukee, WI). The pre-imaging algorithm of the PROBE software automatically adjusted the transmitter and receiver gains and centre frequency. The local magnetic field homogeneity was optimised with the three-plane auto-shim procedure with linear gradient shimming, and the flip angle of the third water-suppression pulse was adjusted for chemical shift water suppression (CHESS) before PRESS acquisition. Each spectrum was automatically fitted to four peaks corresponding to the levels of N-acetylaspartate (NAA) (2.02 ppm), total creatine (Cr) (3.03 ppm), choline-containing compounds (Cho) (3.23 ppm) and myo-inositol (mI) (3.56 ppm). Metabolite intensity ratios of NAA/Cr, Cho/Cr, and mI/Cr were automatically calculated at the end of each PROBE/SV acquisition.

MRS data were post-processed by means of software provided by the manufacturer (GE workstation, ADW 4.4). The ratios of NAA, Cho, and mI to creatine (NAA/Cr, Cho/Cr, mI/Cr, respectively) were assessed. We used the same methods as in our previous paper^21,22^.

### Statistical analysis

Statistical computations were performed using the Statistica PL software package version 13.0, and p < 0.05 was set as a significant level.

The event-related potentials (ERP) values in patients with HT and healthy subjects were compared using the Student T-test. Additionally, in order to assess sensitivity, specificity, and accuracy of ERP in distinguishing patients with HT and normal controls, receiver-operating characteristic (ROC) analysis was performed for ERP showing the most significant differences between these two groups. The rate of accuracy was based on the area under the ROC curve. Associations between event-related potentials and MRS measurements in patients with HT were assessed using Pearson’s correlation coefficient.

### Ethical approval

All procedures performed in studies involving human participants were in accordance with the ethical standards of the institutional and/or national research committee and with the 1964 Helsinki declaration and its later amendments or comparable ethical standards. Approval for this research was given by the Commission of Ethics at the Wrocław Medical University (number of permission: KB-313/2013). Informed consent was obtained from each patient to participate in this study. This article does not contain any studies with animals performed by any of the authors.

### Informed consent

Informed consent was obtained from all individual participants included in the study.

## Results

### Endocrinological, neurological examination and cognition

The mean value for TSH was 1.94 ± 1.08 UIU/ml (normal value 0.35–5.6 UIU/ml), fT3 2.94 ± 0.40 pg/ml (normal value 2.5–3.9 pg/ml), fT4 1.03 ± 0.16 ng/dl (normal value 0.61–1.12 ng/dl), TPOAb 482 ± 371 IU/ml, TGAb 133 ± 238 IU/ml (Table 1). The disease duration ranged from 7 to 41 years, and the average disease duration was 39 months (for women 37 months and for men 14 months).

**Table 1:** Demographic and laboratory characteristics of HT patients and control group.

	HT patients (n = 68)	Control group (n = 45)	*p* value
Sex (female/male)	59/9	39/6	0.461
Age (years)	44.3	44.1	0.367
Education (years)	11.5	12	0.275
BMI (kg/m^2^)	24.6 ± 3.1	24.7 ± 3.3	0.402
TSH (UIU/ml)	1.94 ± 1.08	1.71 ± 1.1	0.167
fT3 (PG/ml)	2.94 ± 0.40	2.91 ± 0.46	0.225
fT4 (NG/dl)	1.03 ± 0.16	1.06 ± 0.23	0.243
TGAb (IU/ml)	133 ± 238	–	–
TPOAb (IU/ml)	482 ± 371	–	–
dose of levothyroxine (µg/day)	64 ± 37	–	–

At diagnosis 45 patients (38 women, 7 men) have overt hypothyroidism and 23 (21 women, 2 men) have subclinical hypothyroidism.

The neurological examination and the results of the MoCA, TMT-A, SDMT and CDT were normal in all patients (100%).

### Event-related potentials

The mean values for the latency of N200 and P300 potentials recorded from all electrodes (Fz, Cz, Pz) were significantly longer in HT patients than in the control group (p < 0.001). The mean amplitude of P300 potentials recorded from all electrodes was significantly lower in HT patients than in the control (Table 2).

**Table 2:** The mean value of the latency (ms) and amplitude (uV) of N200 and P300 potentials in patients with HT and in the control group.

ERP	HT patients (n = 68)		Control group (n = 45)		*p*-value
	Mean	SD	Mean	SD	
**Latency (ms)**					
N200 Fz	246.5	34.7	211.5	20.2	< 0.001
Cz	245.6	33.7	211.3	20.6	< 0.001
Pz	245.9	32.9	212.1	19.7	< 0.001
P300 Fz	351.6	22.5	322.8	21.2	< 0.001
Cz	352.1	22.7	323.5	22.3	< 0.001
Pz	353.4	22.4	325.6	22.9	< 0.001
**Amplitude (uV)**					
N200 Fz	3.51	2.83	4.00	2.44	0.36
Cz	3.39	2.23	3.44	2.53	0.92
Pz	2.99	2.18	3.06	1.89	0.86
P300 Fz	5.57	3.68	8.04	3.93	0.002
Cz	5.63	3.72	8.02	3.73	0.001
Pz	5.03	3.59	7.86	3.81	0.0001

*ERP* event-related potential, *HT* Hashimoto’s thyroiditis, *SD* standard deviation, *Fz* frontal region, *Cz* central region, *Pz* parietal region.

### Event-related potentials in HT patients and control group according to age

In order to compare HT patients to control group according to their age, HT patients and CG were divided into three groups as follows: 20–39 years, 40–59 years and over 60 years old.

HT patients showed a statistically significant longer N200 and P300 latencies and lower P300 amplitudes from all electrodes in all aged groups compared to the control group (Table 3).

**Table 3:** Mean values and standard deviations (SD) of N200 and P300 parameters in HT patients depending on their age.

Age	20–39 years			40–59 years			> 60 years		
No of patients	HT (n = 26)	CG (n = 17)	p-value	HT (n = 30)	CG (n = 18)	p-value	HT (n = 12)	CG (n = 10)	p-value
	Mean (SD)	Mean (SD)		Mean (SD)	Mean (SD)		Mean (SD)	Mean (SD)	
**Latency (ms)**									
N200 Fz	231.3 (31.9)	204.1 (19.1)	< 0.001	249.3 (30.5)	212.8 (20.5)	< 0.001	272.6 (35.2)	223.0 (17.3)	< 0.001
Cz	231.6 (31.9)	201.4 (17.4)	< 0.001	248.0 (29.4)	213.7 (20.7)	< 0.001	270.2 (34.3)	225.1 (18.1)	0.001
Pz	233.7 (30.0)	202.7 (18.3)	< 0.001	247.8 (31.5)	214.1 (18.3)	< 0.001	267.9 (32.4)	225.1 (17.5)	0.001
P300 Fz	338.7 (21.8)	312.8 (19.8)	< 0.001	354.5 (17.3)	323.4 (19.9)	< 0.001	372.1 (18.6)	340.5 (14.4)	< 0.001
Cz	337.9 (20.6)	313.8 (19.6)	< 0.001	355.5 (16.7)	324.7 (24.2)	< 0.001	374.7 (19.5)	339.6 (13.5)	< 0.001
Pz	341.5 (19.8)	318.1 (22.5)	< 0.001	355.3 (16.9)	325.2 (24.7)	< 0.001	374.5 (24.7)	340.9 (11.8)	< 0.001
**Amplitude (uV)**									
N200 Fz	4.68 (3.72)	5.17 (2.95)	0.65	2.88 (1.76)	3.11 (1.51)	0.65	2.58 (1.97)	3.56 (2.23)	0.28
Cz	4.14 (2.85)	4.24 (3.61)	0.92	2.95 (1.65)	2.83 (1.51)	0.81	2.89 (1.56)	3.16 (1.35)	0.68
Pz	3.31 (2.73)	3.34 (2.78)	0.96	2.80 (1.87)	2.67 (0.99)	0.80	2.76 (1.53)	3.24 (1.10)	0.42
P300 Fz	5.65 (2.83)	9.86 (4.75)	< 0.001	6.03 (4.58)	6.12 (2.56)	0.96	5.41 (2.93)	8.39 (2.87)	0.02
Cz	5.38 (2.44)	9.96 (4.56)	< 0.001	6.15 (4.91)	6.31 (2.53)	0.89	4.90 (2.48)	7.65 (2.35)	0.01
Pz	4.81 (2.57)	10.2 (4.43)	< 0.001	5.76 (4.55)	6.85 (2.63)	0.94	3.68 (2.33)	7.5 (2.18)	< 0.001

*CG* control group, *HT* Hashimoto’s thyroiditis, *SD* standard deviation, *Fz* frontal region, *Cz* central region, *Pz* parietal region.

### Event-related potentials in HT patients depending on the length of the disease

In order to compare HT patients to the control group according to the duration of the disease, HT patients were divided into four groups as follows: duration of the disease up to 10 years (19 subjects), duration of the disease 11–20 years (20 subjects), duration of the disease 21–30 years (16 subjects), duration of the disease over 30 years (13 subjects).

HT patients showed a statistically significant longer N200 and P300 latencies and lower P300 amplitudes from all electrodes in all groups compared to the control group (Table 4).

**Table 4:** Mean values and standard deviations (SD) of N200 and P300 parameters in HT patients depending on the duration of the disease.

Duration of the disease	N/A	< 10 years		11–20 years		21–30 years		> 30 years	
No of patients	CG (n = 45)	HT (n = 19)	HT vs CG	HT (n = 20)	HT vs CG	HT (n = 16)	HT vs CG	HT (n = 13)	HT vs CG
	Mean (SD)	Mean (SD)	p-value	Mean (SD)	p-value	Mean (SD)	p-value	Mean (SD)	p-value
**Latency (ms)**									
N200 Fz	211.5 (20.2)	232.4 (27.9)	0.001	234.2 (30.1)	< 0.001	256.9 (34.3)	< 0.001	273.4 (33.8)	< 0.001
Cz	211.3 (20.6)	232.3 (28.9)	0.001	234.5 (29.1)	< 0.001	255.9 (33.4)	< 0.001	270.4 (32.8)	< 0.001
Pz	212.1 (19.7)	235.2 (25.4)	0.002	233.4 (28.9)	0.001	256.5 (36.4)	< 0.001	268.0 (31.0)	< 0.001
P300 Fz	322.8 (21.2)	336.3 (21.5)	0.02	347.7 (16.7)	< 0.001	358.9 (19.7)	< 0.001	370.8 (18.5)	< 0.001
Cz	323.5 (22.3)	336.4 (21.7)	0.04	347.6 (14.2)	< 0.001	359.3 (20.0)	< 0.001	373.3 (19.3)	< 0.001
Pz	325.6 (22.9)	340.6 (20.2)	0.01	348.0 (14.1)	< 0.001	359.1 (20.6)	< 0.001	373.4 (24.0)	< 0.001
**Amplitude (uV)**									
N200 Fz	4.00 (2.44)	4.66 (3.88)	0.41	3.58 (2.65)	0.54	2.93 (1.72)	0.11	2.46 (1.93)	0.14
Cz	3.44 (2.53)	4.04 (2.92)	0.42	3.39 (2.19)	0.94	3.02 (1.79)	0.54	2.92 (1.52)	0.48
Pz	3.06 (1.89)	3.22 (2.39)	0.77	3.03 (2.49)	0.96	2.88 (2.16)	0.75	2.73 (1.49)	0.57
P300 Fz	8.04 (3.93)	6.04 (3.19)	0.05	5.89 (4.58)	0.05	5.55 (3.88)	0.03	5.50 (2.82)	0.03
Cz	8.02 (3.73)	5.69 (2.66)	0.01	5.94 (4.79)	0.05	5.71 (4.39)	0.05	5.02 (2.41)	< 0.001
Pz	7.86 (3.81)	4.95 (2.54)	0.01	5.02 (4.26)	0.001	6.16 (4.48)	0.01	3.86 (2.21)	< 0.001

*CG* control group, *HT* Hashimoto’s thyroiditis, *SD* standard deviation, *Fz* frontal region, *Cz* central region, *Pz* parietal region.

### ROC analysis for event-related potentials

**Figure 3:**
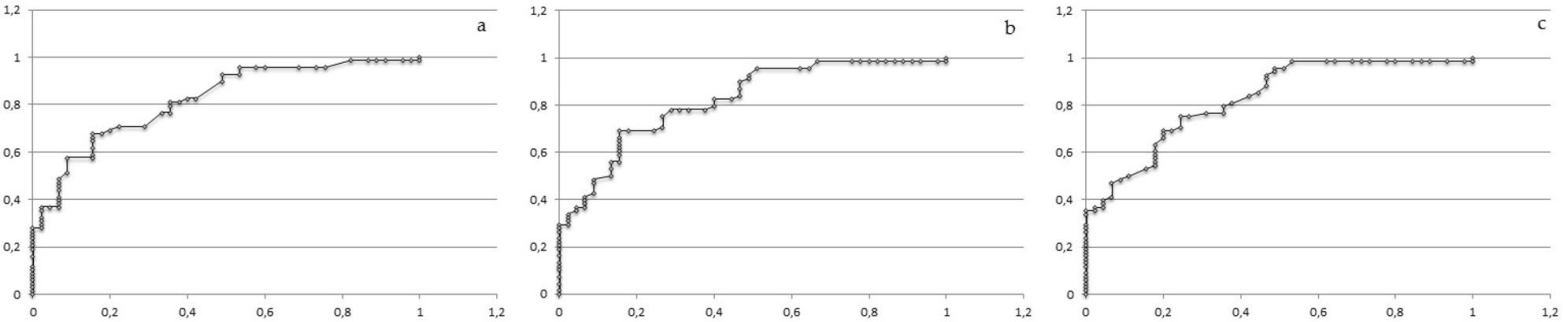
**(a–c)** The receiver-operating characteristic (ROC) curves for the N200 latency. **(a)** Fz N200. **(b)** Cz N200. **(c)** Pz N200. *Fz* frontal region, *Cz* central region, *Pz* parietal region; X axis—specificity; Y axis—sensitivity.

**Figure 4:**
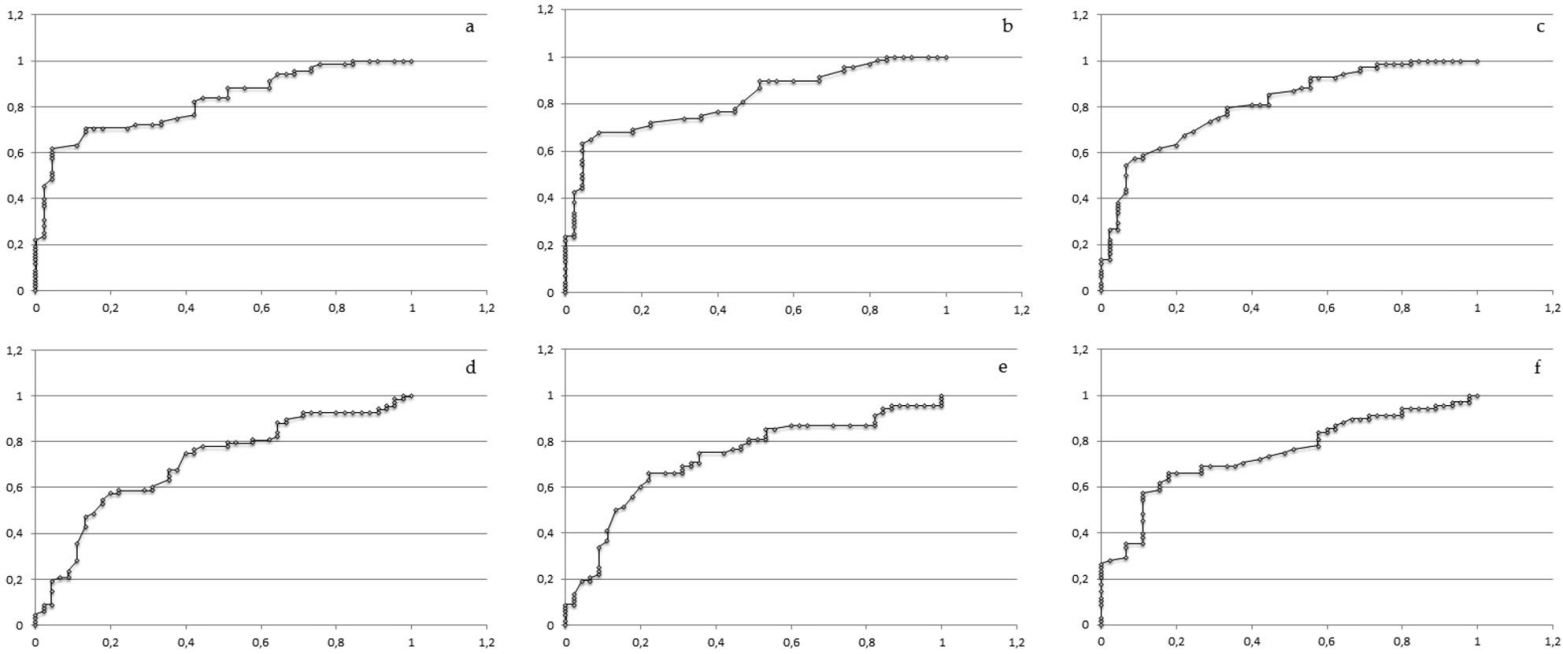
**(a–f)** The receiver-operating characteristic (ROC) curves for the P300 components. **(a)** Fz latency. **(b)** Cz latency. **(c)** Pz latency. **(d)** Fz amplitude. **(e)** Cz amplitude. **(f)** Pz amplitude. *Fz* frontal region, *Cz* central region, *Pz* parietal region; X axis—specificity; Y axis—sensitivity;

ROC analysis was performed separately for the N200 and P300 ERP components (Table 5, Figs. 3, 4). Both ROC curves demonstrated good diagnostic accuracy with the area under the curve above 0.80 for the latency value of N200 (Fig. 3) and for the latency value of P300 (Fig. 4a–c). ROC analysis for the value of the amplitude showed good accuracy with the area under the curve above 0.70 only for P300 components (Fig. 4d–f).

**Table 5:** Results of ROC analyses for the N200 and P300 ERP components.

ERP	Cut point values	Specificity	Sensitivity	Accuracy
**Latency (ms)**				
N200 Fz	225	0.84	0.67	0.82
Cz	224	0.84	0.66	0.82
Pz	221	0.84	0.67	0.82
P300 Fz	343	0.86	0.71	0.82
Cz	344	0.91	0.67	0.82
Pz	349	0.91	0.57	0.81
**Amplitude (uV)**				
P300 Fz	4.71	0.8	0.57	0.71
Cz	5.23	0.77	0.66	0.73
Pz	5.32	0.82	0.66	0.75

*ERP* event-related potential, *Fz* frontal region, *Cz* central region, *Pz* parietal region.

### Correlations of ERP and MRS measurements

**Figure 5:**
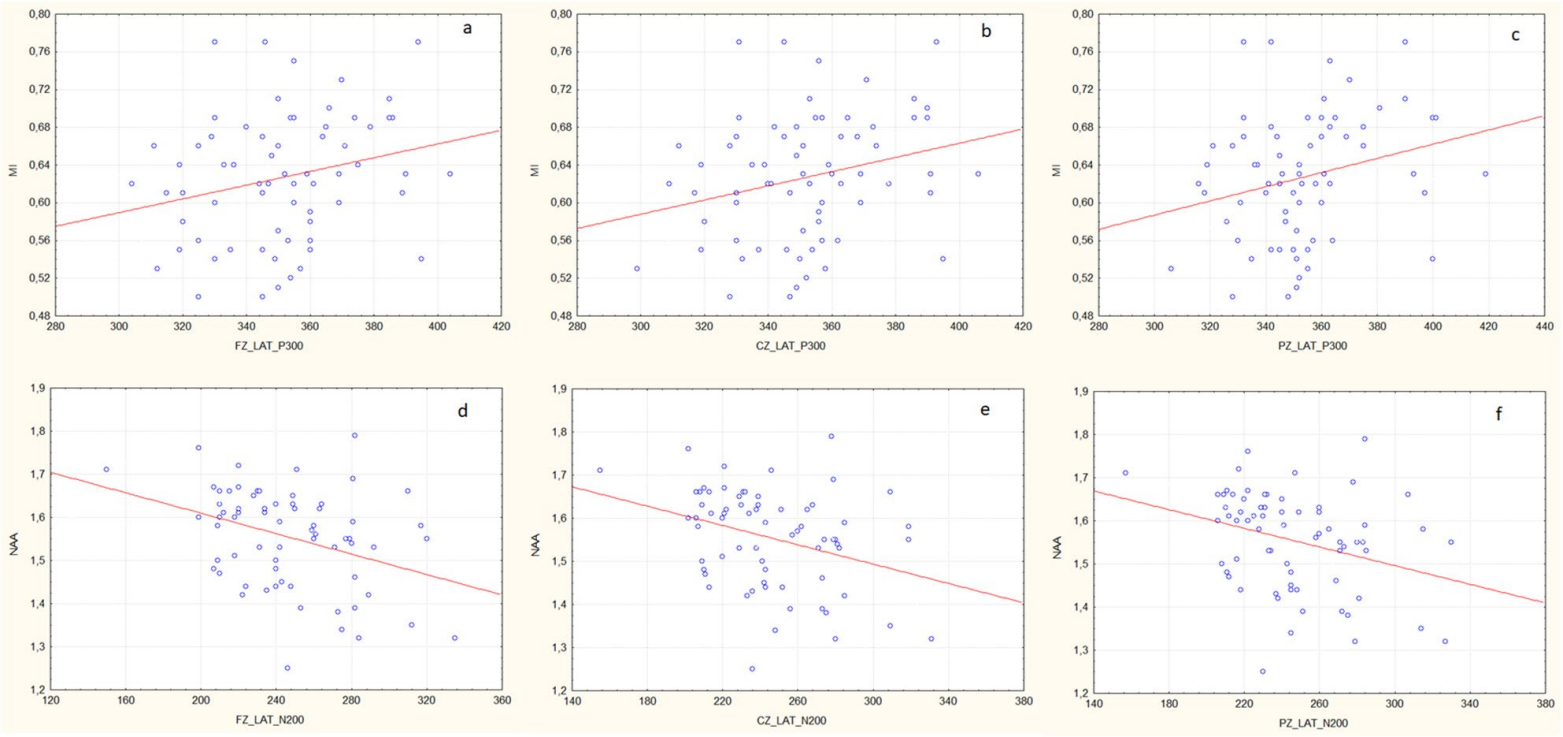
**(a–f)** Correlations of ERP and MRS measurements in the posterior cingulate gyrus (PCG) region. **(a)** A significantly positive correlation between mI/Cr ratio and P300 Fz latency (r = 0.224, p = 0.045). **(b)** A significantly positive correlation between mI/Cr ratio and P300 Cz latency (r = 0.225, p = 0.035). **(c)** A significantly positive correlation between mI/Cr and P300 Pz latency (r = 0.252, p = 0.038). **(d)** A significantly negative correlation between NAA/Cr ratio and N200 Fz latency (r = -0.358, p = 0.003). **(e)** A significantly negative correlation between NAA/Cr ratio and N200 Cz latency (r = − 0.329, p = 0.006). **(f)** A significantly negative correlation between NAA/Cr ratio and N200 Pz latency (r = − 0.311, p = 0.01). *Fz* frontal region, *Cz* central region, *Pz* parietal region, *NAA* *N*-acetylaspartate, *Cr* creatine, *mI* myo-inositol.

There was a significant positive correlation between the mI/Cr ratio in the PCG area and P300 latencies (Fz, Cz, Pz) (r = 0.224, p = 0.045; r = 0.225, p = 0.035; r = 0.252, p = 0.038, respectively) (Fig. 5a–c). NAA/Cr ratio in the PCG region showed significant negative correlations with all N200 latencies (Fz, Cz, Pz) (r = -0.358, p = 0.003; r = − 0.329, p = 0.006; r = − 0.311, p = 0.01, respectively) (Fig. 5d–f).

There were no significant correlations between ERP and metabolite ratios in the PWM region.

There were no statistically significant differences between ERP and MRS parameters in patients with different initial diagnosis—overt hypothyroidism and subclinical hypothyroidism.

### Correlations of EPR and thyroid hormones

There were no statistically significant correlations between the mean ERP parameters, thyroid hormones levels, and TGAb and TPOAb either.

### Correlations of MRS measurements and thyroid hormones

The MRS parameters showed no statistically significant correlations neither with thyroid hormones levels nor TGAb and TPOAb .

## Discussion

The clinical manifestations of autoimmune thyroiditis are not specific and are often low. However, many patients report numerous subjective complaints that have a significant impact on their quality of life^5,7^. In *The Colorado Thyroid Disease Prevalence Study* conducted in 2000, 18,750 people with compensated thyroid function were identified among 25,000 people in the general population, 114 patients with overt hypothyroidism and over 2300 patients with subclinical hypothyroidism^23^. It was shown that 12.1% of patients with euthyroid thyroid disease and 13.7% of patients with subclinical hypothyroidism reported constellations of symptoms and complaints found in 16.6% of patients with overt hypothyroidism. Among the reported complaints, memory disorders (24%), slow thinking (22%) and fatigue (18%) were most frequently mentioned^23^. Sharma et al.^24^ analysed the clinical profile of 13 patients with diagnosed Hashimoto encephalopathy (HE) for the complaints reported by patients. Most often, patients complained of memory and concentration disorders and cognitive impairment (76.9%), sleep disorders (69.21%) including hypersomnia (46.2%) or insomnia (23%). The pathomechanism of these symptoms has not yet been clarified.

Up to now, ERP and MRS studies have not been conducted in large groups of patients with hormonally compensated Hashimoto's thyroiditis. It is worth mentioning that Bladowska et al. assessed metabolic alterations within the normal appearing brain in 55 subjects with HT using MRS and they also correlated MRS measurements with hormonal concentrations^22^. They found significantly decreased NAA/Cr ratios in PCG and parietal white matter (PWM) in HT patients. Their results also showed significant positive correlations between the NAA/Cr ratio and fT3 level. Since the reduction of NAA/Cr ratios suggests a decrease of neuronal activity, the study by Bladowska et al. indicated the early cerebral metabolic disturbances associated with Hashimoto’s thyroiditis and thus suggesting that MRS could be a sensitive marker of cognitive decline in presymptomatic subjects^22^. A few, isolated studies describe cases of a rare complication of Hashimoto's encephalopathy^25^. A report describing the results of MRS in a 52-year-old woman with HE was published by Chinese authors. In the patient's brain they found a reduced level of NAA and mI, in addition—diffuse, atypical inflammatory changes^26^. In a SPECT study, features of diffuse hypoperfusion were described in patients with HE^27^.

We have found significantly longer latencies in the N200 and P300 components in HT patients, and reduced P300 wave amplitudes in all leads. During the ERP and brain imaging examinations, all patients remained in euthyreosis and none of them had signs of CNS damage in the neurological examination. People with conditions that could affect brain bioelectrical activity and/or damage the nervous system were excluded from the analysis.

Changes in ERP parameters are a measure of the depth of cognitive impairment, however, their bundles with the severity and extent of changes in the CNS remain ambiguous^14^. Despite specific CNS structures generating individual ERP components such as prefrontal, temporo-parietal cortex and hippocampal region, their result is considered to reflect the overall functioning of extensive neural networks^14,16^. Analysing the correlations of various neuropsychological tests with ERP, no specific tests were identified that would best correlate with ERP results. The results of various authors are ambiguous and most often relate to tests used to assess global cognitive functioning^28,29^.

Interestingly, we found significant correlations of ERP and MRS measurements. NAA/Cr ratio in the PCG region showed significant negative correlations with all N200 latencies. NAA is a neuronal marker, thus its concentration correlates with neuronal density and neuronal function. As mentioned above, the decrease of NAA/Cr ratio may indicate a reduction of neuronal activity. We observed that the lower NAA/Cr ratios in the PCG region, the longer are the values for the latency of N200 potentials recorded from all electrodes. Furthermore, it should be stressed that the PCG is a very special brain region involved in cognitive function. The PCG is a part of the memory-related default mode network (DMN), an important system of interacting brain regions associated with Alzheimer's Disease (AD). Disorders of the DMN are considered to be an important feature of AD. Moreover, the PCG cortex plays important roles in episodic memory, spatial attention, self-evaluation, and other cognitive functions^30^. Studies have been suggested that abnormal outgoing connections from the PCG cortex to other brain areas may be an imaging biomarker for the early stages of cognitive impairment, especially AD. The most important goal is to perform medical interventions as early as possible. It has been reported that early appropriate management of risk patients can not only effectively delay the progression of the disease and extend survival, but also improve the quality of life of the affected subjects and reduce the burden on the whole of society^30^.

According to these findings our results suggest that both the decrease of NAA/Cr ratio and the longer values for the latency of N200 potentials may indicate the functional deterioration in the PCG cortex.

Furthermore, there was also a significant positive correlation between the mI/Cr ratio in the PCG area and P300 latencies. It is known that mI is an in vivo biomarker of glial cell inflammation and activation. Increased mI levels are thought to be connected with CNS inflammation and gliosis, which may be responsible for neuronal dysfunction^21,22^. It should be emphasised that we did not observe any significant correlations between ERP and metabolite ratios in the white matter of the PWM region, which means that functional impairment may begin in the cortex. Thus early functional disorders can be evaluated with assessing bioelectrical activity of cortical areas of the brain using the cognitive event-related potentials.

Our findings showing the significant relationship between the increased mI/Cr ratio in the PCG cortex and the longer values for the latency of P300 potentials are in accordance with our other results described above, suggesting that MRS and ERP may serve as a useful tool in indicating the risk group of patients prone to developing cognitive impairment and thus to be taken care of as soon as possible.

Although studies have shown an increased risk of cognitive impairment in HT patients, even in the euthyroid state, the mechanisms behind it remain unclear^3,8^. Long-term potentiation in the Schaffer collateral-CA1 pathway of the hippocampus may to play a key role in the development of these disorders. In a study on an experimental model of euthyroid HT mice, abnormalities in synaptic plasticity of the Schaffer collateral-CA1 synapses in the hippocampus, worse memory and spatial learning were shown. In transmission electron microscopy the synapses and astrocytes in the hippocampus was damaged. The elements in glutamate-glutamine circulation located in astrocytes, were downregulated and elevated levels of glutamate in the hippocampus was observed^30,31^.

Other studies suggested that inflammation characterised by elevated cytokines plays a key role in the pathogenesis of cognitive impairment in HT^7,24^. Previous studies have shown increased production of cytokines, including interleukin-1β, interleukin-6, tumour necrosis factor alpha and monocyte chemotactic protein-1 in patients with HT. Vitamin D participates in modulating the secretion of cytokines and these results suggest that vitamin D deficiency may play a key role in the cognitive impairment of HT patients^7^. Similar conclusions can be drawn from the study by Cai et al., who demonstrated astrocyte and microglia activation with increased expression of proinflammatory cytokine genes in the frontal cortex on experimental models of an HT mouse, which influenced the serotonin system in this region of the brain^32^. No significant direct effect of the anti-thyroid antibodies themselves on the structures of the central nervous system has been demonstrated, although some researchers have suggested that they may weaken the integrity of the myelin sheath and affect many neurotransmitters^3^.

As mentioned above, we observed significant metabolic changes in the PCG region as well as significant correlations between MRS parameters and ERP. Our results indicate that there are alterations in the important brain region involved in the cognition process, thus obviously leading to cognitive impairment in HT patients. However, it should be stressed that the advanced MR techniques, although able to show even subtle preclinical changes, do not allow us to speculate as to which mechanism could be responsible for brain injury and finally cognitive impairment, as their results are non-specific. By using the MRS method it is possible to assess the metabolic impairment in specific brain regions such as the PCG region, which has already been proven to be associated with cognition, but these alterations may not presume the underlying mechanism^22,33^.

The limitations include conducting the study in only one time point in HT patients, which is known to have a fluctuating hormonal course. However, our previous report was the first pilot study in the world literature whose findings might encourage further research. Our future studies will focus on monitoring ERP and MRS parameters in the course of the disease and analysing the relationship between cognitive impairment and their predictive cognitive outcome of HT patients.

## Conclusions

To our knowledge, this is the first study examining ERP and MRS findings in euthyroid patients with HT without cognitive impairment.

We found significant correlations of ERP and MRS measurements in the PCG region. The NAA/Cr ratio showed significant negative correlations with all N200 latencies, while the mI/Cr ratio revealed a significant positive correlation with P300 latencies, which means that decreased values of the N200 and P300 latencies are associated with metabolic alterations in the PCG cortex. Both findings may indicate important functional changes in the cortex of the PCG which can be assessed with ERP as well as MRS measurements. Therefore functional disorders observed in the PCG cortex may suggest some brain dysfunction in neurologically asymptomatic HT patients. The mandatory goal of everyday clinical practice is to assess the group of HT subjects who are at risk of cognitive disorders and accordingly they need early and dedicated medical care in order to achieve cerebral function recovery or at least to stop progression of brain damage in the course of HT.

The lack of significant differences between the ERP and MRS parameters in HT patients with different initial diagnosis may indicate the influence of the autoimmune process itself, and not of the severity of pre-therapy thyroid dysfunction, on the development of cognitive impairment.

ERPs undergo significant changes in patients with HT and may, apart from neuroimaging studies, constitute an important element of recognising and monitoring cognitive functions in this group of patients. The significant correlations we observed with the MRS parameters justify further research in this area.

## References

[1] Caturegli P, De Remigis A, Rose NR (2014). Hashimoto thyroiditis: Clinical and diagnostic criteria. Autoimmun. Rev..

[2] Snijders G, de Witte L, van den Berk D (2020). No association between anti-thyroidperoxidase antibodies and bipolar disorder: A study in the Dutch Bipolar Cohort and a meta-analysis. Psychoneuroendocrinology..

[3] Leyhe T, Müssig K (2014). Cognitive and affective dysfunctions in autoimmune thyroiditis. Brain Behav. Immun..

[4] Broniarczyk-Czarniak M (2017). The prevalence of psychiatric disorders in patients with Hashimoto’s thyroiditis: A literature review. Psychiatria.

[5] Carta M (2015). A case control study on psychiatric disorders in Hashimoto disease and Euthyroid Goitre: Not only depressive but also anxiety disorders are associated with thyroid autoimmunity. Clin. Pract. Epidemiol. Ment. Health..

[6] Dejanovic M, Ivetic V, Nestorivic V, Milanowic Z, Eric M (2017). The value of P300 event related potentials in the assessment of cognitive function in subclinical hypothyroidism. Miner. Endocrinol..

[7] Xu J, Xiang-Yu Z, Hui S (2018). Low vitamin D levels are associated with cognitive impairment in patients with Hashimoto thyroiditis. BMC Endocr. Disord..

[8] Djurovic M, Pereira AM, Smit JWA (2018). Cognitive functioning and quality of life in patients with Hashimoto thyroiditis on long-term levothyroxine replacement. Endocrine.

[9] Polich J (2007). Updating P300: An integrative theory of P3a and P3b. Clin. Neurophysiol..

[10] Gupta RS, Kujawa A, Vago DR (2019). The neural chronometry of threat-related attentional bias: Event-related potential (ERP) evidence for early and late stages of selective attentional processing. Int. J. Psychophysiol..

[11] Duncan C (2009). Event-related potentials in clinical research: guidelines for eliciting, recording, and quantifying mismatch negativity, P300, and N400. Clin. Neurophysiol..

[12] Wojcik G (2018). New protocol for quantitative analysis of brain cortex electroencephalographic activity in patients with psychiatric disorders. Front. Neuroinform..

[13] Markiewicz R, Masiak J (2019). Evaluation of cognitive deficits in schizophrenia using event-related potentials and rehabilitation influences using EEG biofeedback in patients diagnosed with schizophrenia. Psychiatr. Pol..

[14] Patel S, Azzam P (2005). Characterization of N200 and P300: Selected studies of the event-related potentials. Int. J. Med. Sci..

[15] Goodin D, Desmedt J, Maurer K, Nuwer J (2014). IFCN recommended standards for long-latency auditory event-related potentials. Report of an IFCN committee. Clin. Neurophysiol..

[16] Suchotzki K, Crombez G, Smulders FT, Meijer E, Verschuere B (2015). The cognitive mechanisms underlying deception: An event-related potential study. Int. J. Psychophysiol..

[17] Rönnberg J (2016). Hearing impairment, cognition and speech understanding: Exploratory factor analyses of a comprehensive test battery for a group of hearing aid users, the N200 study. Int. J. Audiol..

[18] Waliszewska-Prosół M, Nowakowska-Kotas M, Kotas R, Bańkowski T, Pokryszko-Dragan A, Podemski R (2018). The relationship between event-related potentials, stress perception and personality type in patients with multiple sclerosis without cognitive impairment: A pilot study. Adv. Clin. Exp. Med..

[19] Chalah M, Kauv P, Créange A, Hodel J, Lefaucheur JP, Ayache SS (2019). Neurophysiological, radiological and neuropsychological evaluation of fatigue in multiple sclerosis. Mult. Scler. Relat. Disord..

[20] Artemiadis A, Anagnostouli M, Zalonis I, Chairopoulos K, Triantafyllou N (2018). Structural MRI correlates of cognitive event-related potentials in multiple sclerosis. J. Clin. Neurophysiol..

[21] Bladowska J (2013). Evaluation of early cerebral metabolic, perfusion and microstructural changes in HCV-positive patients: A pilot study. J. Hepatol..

[22] Bladowska J, Waliszewska-Prosół M, Ejma M, Sąsiadek M (2019). The metabolic alterations within the normal appearing brain in patients with Hashimoto's thyroiditis are correlated with hormonal changes. Metab. Brain Dis..

[23] Canaris G, Manowitz N, Mayor G, Ridgway EC (2000). The Colorado thyroid disease prevalence study. Arch. Intern. Med..

[24] Sharma P, Javali M, Mahale R, Madhusudhan B, Abdul A (2015). Hashimoto encephalopathy: A study of the clinical profile, radiological and electrophysiological correlation in a Tertiary Care Center in South India. J. Neurosci. Rural Pract..

[25] Churilov LP, Sobolevskaia PA, Stroev Y (2019). Thyroid gland and brain: Enigma of Hashimoto's encephalopathy. Best Pract. Res. Clin. Endocrinol. Metab..

[26] Su TH, Jin E, He W (2011). Hashimoto encephalopathy: a case report with proton MR spectroscopic findings. Chin Med. J. (Engl).

[27] Kaya M, Cermik TF, Bede D (2007). Assessment of alterations in regional cerebral blood flow in patients with hypothyroidism due to Hashimoto’s thyroiditis. J. Endocrinol. Invest..

[28] Oishi M, Mochizuki Y, Takasu T (1997). Differences in P300 latency in two types of leukoaraiosis. J. Neurol..

[29] Taghavy A, Hamer H (1995). Symptomatic and asymptomatic high-grade unilateral internal carotid artery stenosis: Scalp topography of event-related potentials (P300) and psychometric testing. Electroencephalogr. Clin. Neurophysiol..

[30] Yu E, Liao Z, Zhang Q (2017). Directed functional connectivity of posterior cingulate cortex and whole brain in Alzheimer's disease and mild cognitive impairment. Curr. Alzheimer Res..

[31] Wang, N. *et al*. Hashimoto's thyroiditis induces hippocampus-dependent cognitive alterations by impairing astrocytes in euthyroid mice. *Thyroid*. **12** (2020). 10.1089/thy.2020.0139 32907517

[32] Cai YJ (2018). Hashimoto's thyroiditis induces neuroinflammation and emotional alterations in euthyroid mice. J. Neuroinflamm..

[33] Faghihi R, Zeinali-Rafsanjani B, Mosleh-Shirazi MA, Saeedi-Moghadam M, Lotfi M, Jalli R (2017). Magnetic resonance spectroscopy and its clinical applications: A review. J. Med. Imaging Radiat. Sci..

